# Retraction: A Systematic Review of Accelerated Intermittent Theta Burst Stimulation (iTBS) Versus Daily Repetitive Transcranial Magnetic Stimulation (rTMS) for Treatment-Resistant Depression: Is Shorter Really Better?

**DOI:** 10.7759/cureus.r246

**Published:** 2026-09-17

**Authors:** Ayushi Saxena, Bilal Khan, Rupanshu Rupanshu, Russaal S Mann, Isha Chopra, Abdullah Kilic, Pousette F Hamid

**Affiliations:** 1 Research, California Institute of Behavioral Neurosciences & Psychology, Fairfield, USA; 2 Medicine, St. Martinus University Faculty of Medicine, Willemstad, CUW; 3 Health Sciences, Queen's University, Kingston, CAN; 4 Internal Medicine, St. Martinus University Faculty of Medicine, Willemstad, CUW; 5 Medicine, Vardhman Mahavir Medical College and Safdarjung Hospital, Delhi, IND; 6 Anesthesiology, Pandit Naval Kishore Sharma (PNKS) Government Medical College, Dausa, IND; 7 Internal Medicine, Hackensack University Medical Center, Montclair, USA; 8 Neurology, Ain Shams University, Cairo, EGY; 9 Neurology, California Institute of Behavioral Neurosciences & Psychology, Fairfield, USA

This article has been retracted by the Editor-in-Chief. After publication, concerns were raised about the data reported in the article's evidence tables. Post-publication editorial assessment found that the study characteristics and outcome data presented in those tables are not supported by the publications cited for them, and that the set of included studies cannot be identified from the article as published. The first author has acknowledged the errors; the remaining authors did not respond. The findings and conclusions are therefore unreliable.

